# Cryogenic Ion Vibrational Predissociation (CIVP) Spectroscopy of Aryl Cobinamides in the Gas Phase: How Good Are the Calculations for Vitamin B_12_ Derivatives?

**DOI:** 10.1021/jacs.3c03001

**Published:** 2023-09-01

**Authors:** Alexandra Tsybizova, Lukas Fritsche, Larisa Miloglyadova, Bernhard Kräutler, Peter Chen

**Affiliations:** † Laboratorium für Organische Chemie, ETH Zurich, Vladimir-Prelog-Weg 2, CH-8093 Zurich, Switzerland; ‡ Institute of Organic Chemistry, University of Innsbruck, Innrain 80/82, 6020 Innsbruck, Austria

## Abstract

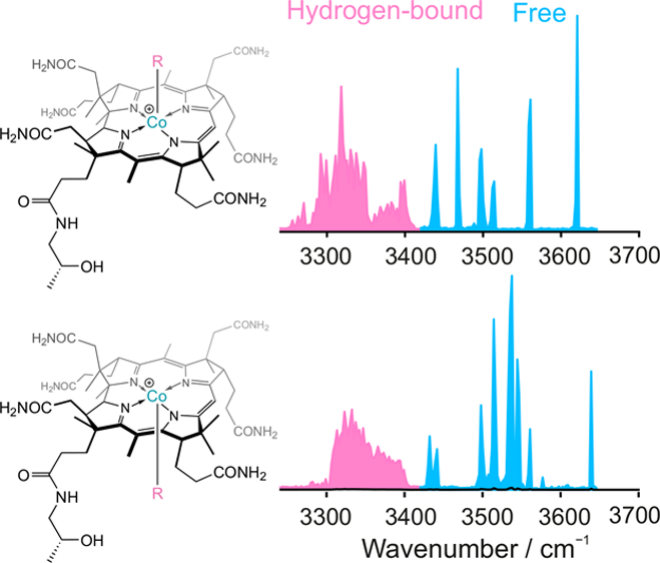

Aryl corrins represent a novel class of designed B_12_ derivatives with biological properties of “antivitamins B_12_”. In our previous study, we experimentally determined bond strength in a series of aryl-corrins by the threshold collision-induced dissociation experiments (T-CID) and compared the measured bond dissociation energies (BDEs) with those calculated with density functional theory (DFT). We found that the BDEs are modulated by the side chains around the periphery of the corrin unit. Given that aryl cobinamides have many side chains that increase their conformational space and that the question of a specific structure, measured in the gas phase, was important for further evaluation of our T-CID experiment, we proceeded to analyze structural properties of aryl cobinamides using cryogenic ion vibrational predissociation (CIVP) spectroscopy, static DFT, and Born–Oppenheimer molecular dynamic (BOMD) simulations. We found that none of the examined DFT models could reproduce the CIVP spectra convincingly; both “static” DFT calculations and “dynamic” BOMD simulations provide a surprisingly poor representation of the vibrational spectra, specifically of the number, position, and intensity of bands assigned to hydrogen-bonded versus non-hydrogen-bonded NH and OH moieties. We conclude that, for a flexible molecule with ca. 150 atoms, more accurate approaches are needed before definitive conclusions about computed properties, specifically the structure of the ground-state conformer, may be made.

## Introduction

Since the isolation of vitamin B_12_ (cyanocobalamin, CNCbl) and the elucidation of its structure,^1^ there has been much progress in the understanding of the chemistry of B_12_-cofactors and the enzymatic reactions depending upon these unique natural corrins.^2−4^ The question of how B_12_-cofactors evolved has also attracted the most fundamental interest.^5^ The evolution of vitamin B_12_ is hypothesized to have occurred in an enigmatic RNA world,^5−7^ in support of early (pre)life processes.^6,8−10^ Interestingly, *S*-adenosylmethionine (SAM), which has a much simpler structure (and may have evolved still earlier), is involved in similar enzymatic radical and methylation reactions as those observed with cobalamins (Cbls).^11−14^ In consequence, the question of the biological advantage offered by the much more complex Cbl molecules has been intriguing. Indeed, the Cbl coenzyme B_12_ serves as an effectively reversible source of the critical adenosyl radical,^15^ and methylcobalamin (MeCbl) is a very versatile catalysts in both heterolytic or homolytic methyl group transfer reactions.^4^ We seek to understand the “engineering” behind the exquisite biological functions of the B_12_-cofactors, and presume, *vide infra*, that the characteristic conserved decoration of Cbls and its cobinamide (Cbi) core part with the peripheral side chains plays a key structural role in this respect. In consequence, it has also become an important challenge to assess the adequacy of modern electronic and computational structural methods in the prediction of the structures and their relative energies.

In our previous study, we measured bond dissociation energies (BDE) in a series of the non-natural aryl cobinamides, which have become available very recently.^16^ Through our experiments, we found that homolytic cleavage of the Co–C_sp2_ bonds in the β-diastereomers of phenyl- and 4-ethylphenylcobinamides results in BDEs of 38.4 and 40.6 kcal mol^–1^, respectively, whereas their corresponding α-diastereomers have larger BDEs, 46.6 and 43.8 kcal mol^–1^, respectively. In view of the approximately *C*_2_-symmetric structure of the corrin ligand core, such large differences were unexpected. Hence, we concluded that noncovalent interactions with the unequal array of peripheral side chains affect the bond strength significantly, and in a computational study of the BDEs, the computed BDEs accordingly depended on the selection of the conformer. Given the discrepancies originating in the comparison of our experiment and DFT results with regard to thermochemical properties, we now investigate structural properties of aryl cobinamides spectroscopically and measure the cryogenic ion vibrational predissociation (CIVP) spectra of the 4-ethylphenylcobinamides (EtPhCbi) in the gas phase. We report a simple experimentally observable metric for the conformation of the peripheral side chains—the number of H-bonded and non-H-bonded NH and OH groups for which practically accessible DFT methods, static or dynamic, perform poorly.

## Methods

### Chemicals

The synthesis of α- and β-4-ethylphenylcobinamides (from here on labeled as αEtPhCbi^+^ and βEtPhCbi^+^, respectively) were described in our previous work.^16^ HPLC-grade methanol and methanol-*d*_4_ were purchased from Sigma-Aldrich and used without further purification.

### Ion Spectroscopy

Electrospray ionization mass spectrometry was used to bring the RCbi^+^ ions of interest into the gas phase. Either methanol or methanol-*d*_4_ (5 μm) solutions of the respective cobinamides were sprayed. The RCbi^+^/RCbi^+^-d_14_ ions were mass-selected and trapped in the cryogenically cooled ICR ion trap that was described in detail previously.^17^ Nitrogen was used as a buffer gas, and the temperature of the trap was kept at 30 K. CIVP spectroscopy was done as previously described.^17,18^

### Computational Studies

Initial RCbi^+^ structures were obtained with the CREST program (version 2.7.1),^19^ which produced up to 300 conformers spanning a range of 6 kcal mol^–1^. The ten best structures for each of the species, identified in the CREST conformational search, were reoptimized with the density functional theory (DFT) calculations with the Gaussian 09 suite,^20^ and the most stable structure after such reoptimization was considered for further analysis. For the geometry optimization, as well as for calculation of the harmonic IR spectra, the BP86 functional,^21,22^ in combination with the def2-TZVP basis set^23^ was used. Density fitting with the Weigend06 (W06) density fitting basis set^24^ was used to improve the performance of the BP86 functional. Dispersion effects were accommodated by Grimme’s D3 dispersion correction with Becke–Johnson damping.^25,26^ DFT calculations were performed both with and without the D3 correction for comparison. The Results section reports the BP86-D3/def2-TZVP harmonic spectra, whereas spectra calculated without D3 correction can be found in the Supporting Information (SI). Frequency analyses further confirmed the nature of the stationary points located as true minima with no imaginary frequencies.

The Born–Oppenheimer molecular dynamics (BOMD) simulations were performed using the CP2K program package.^27^ For faster convergence, the orbital transformation method^28^ was applied. Density functional theory was used as the electronic structure method: BP86 functional with or without Grimme’s dispersion correction and the molecularly optimized double-ζ basis set (MOLOPT-DZVP-SR-GTH)^29^ was applied to all atoms together with the corresponding Goedecker–Teter–Hutter (GTH) pseudopotentials.^30−32^ A time step of 0.5 fs was chosen and the temperature was set to 50 K using a Nosé–Hoover chain thermostat.^33−35^ Starting from optimized geometries of the individual molecules, the systems were equilibrated using the massive thermostat option (individual thermostat for each degree of freedom) for 3 ps. The production simulations were subsequently run for 20 ps. BOMD spectra have been computed with TRAVIS.^36^ The molecular dipole moments were determined from the maximally localized Wannier function centers and the resulting dipole vectors were used to calculate molecular IR spectra as shown previously.^37^

## Results

### Experimental Spectra

**Figure 1:**
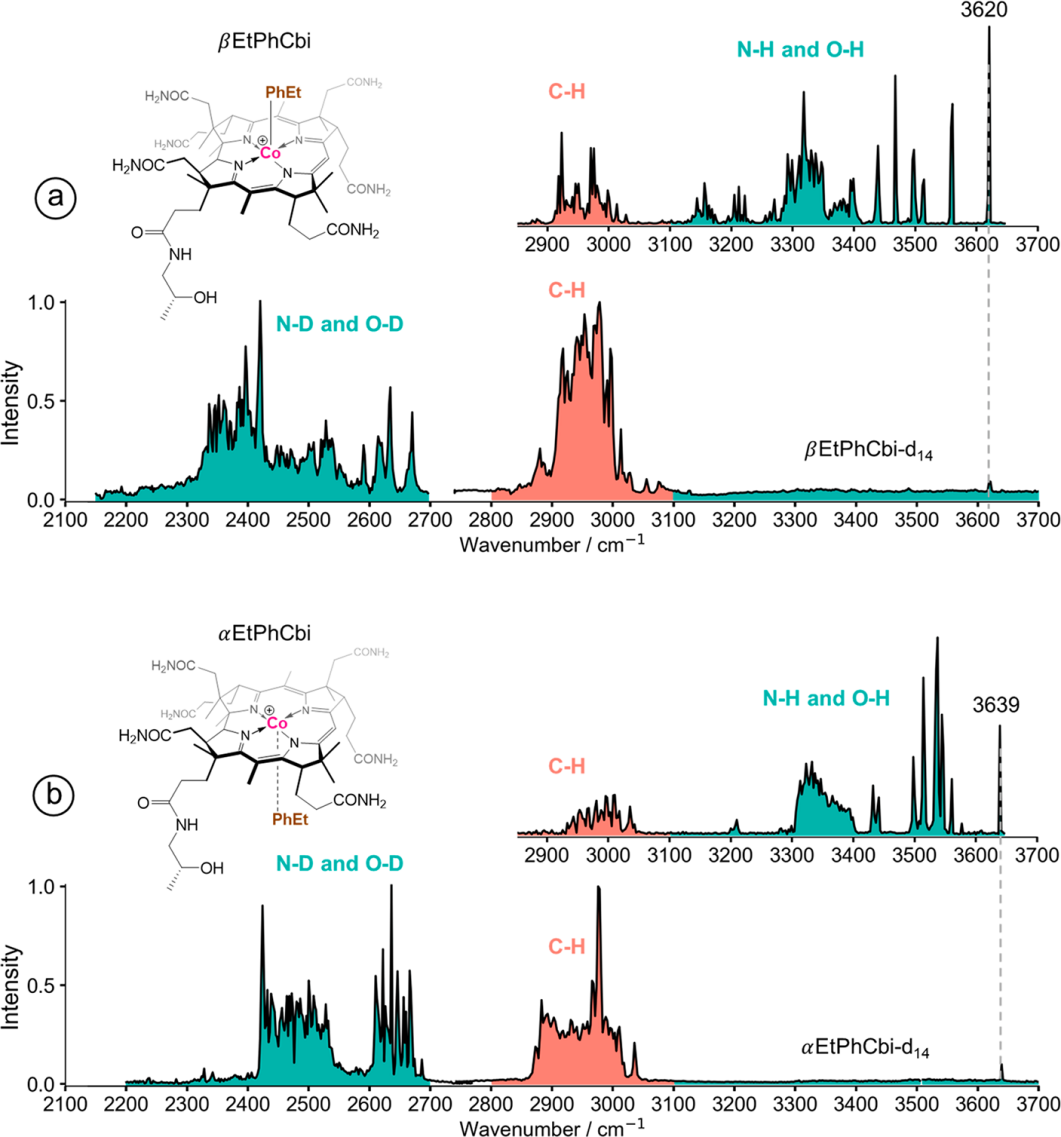
Experimental CIVP spectra of (a) βEtPhCbi and βEtPhCbi^+^-d_14_; (b) αEtPhCbi^+^ and αEtPhCbi^+^-d_14_ recorded with N_2_ as tag.

Figure 1 shows the experimental CIVP spectra of α- and βEtPhCbi^+^. While both diastereomers have similar features in the region 2800–3100 cm^–1^, the spectra look very different in the range 3100–3700 cm^–1^, which can be attributed to the unique conformational structure of the investigated isomers. Noteworthy are sharp intense peaks in the region 3450 to 3700 cm^–1^ for both investigated ions that can be assigned as the free N–H and/or O–H bonds.

To experimentally determine which peaks in the CIVP spectra belong to the (exchangeable) N–H and O–H bonds, we recorded the CIVP spectra of the EtPhCbi^+^ ions sprayed from methanol-d_4_. There are in-total 13 N–H bonds and one O–H bond in each diastereomer that can exchange upon deuteration. The resulting CIVP spectra (Figure 1) of α- and βEtPhCbi^+^-d_14_ show a dramatic red shift of all of the bands higher than 3100 cm^–1^. This confirms that the peaks in the region 3100 to 3600 cm^–1^ all belong to N–H and O–H bonds. Interestingly, for both α- and βEtPhCbi^+^-d_14_, a rather small peak at around 3620 cm^–1^ remains. We attribute this very small peak to a residual N–H or O–H band, presumably due to a small number of isobaric ^1^H_1_^13^C_1_ ions coming through the mass selection.

### Computational Analysis

**Figure 2:**
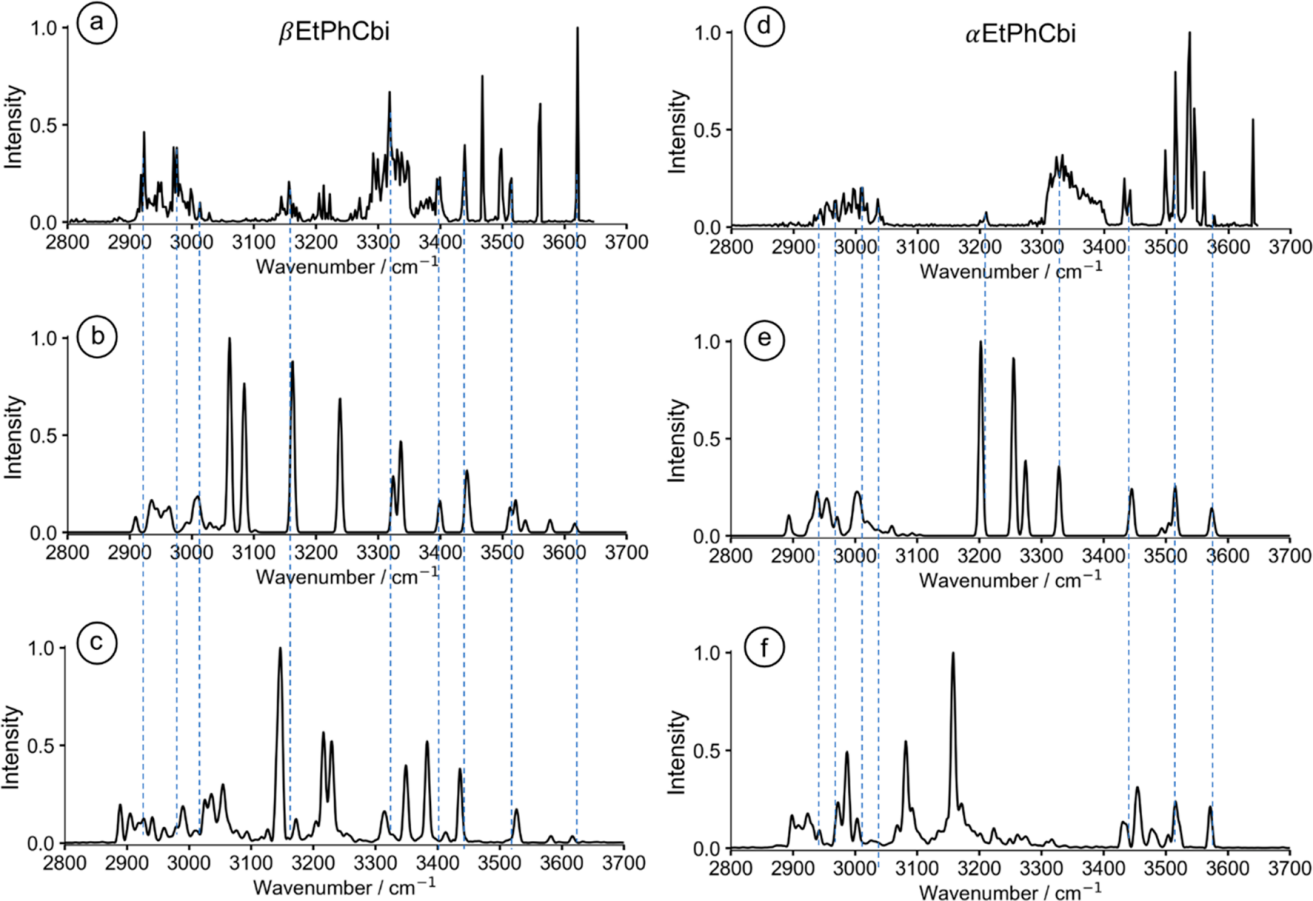
Comparison of the experimental CIVP spectra of (a) β- and (d) α-EtPhCbi with the calculated harmonic IR spectra, (b) and (e), respectively (BP86-D3/def2-TZVP, scaling factor used: 0.99), and BOMD spectra (BP86-D3/DZVP), (c) and (f).

The sharp peaks in the experimental spectra suggest that some of the side chains in both aryl cobinamides remain “free”, *e.g.*, not involved in hydrogen bonding in the lowest energy gas-phase structure. To further characterize our spectra, we proceeded to compute the investigated structures and frequencies. First, we analyzed the experimentally obtained CIVP spectra using DFT with a harmonic approximation. Figure 2 shows a comparison of the CIVP spectra with the BP86-D3/def2-TZVP-calculated harmonic IR spectra.

Even though the experimental and computational spectra correlate well enough in the congested C–H stretching region, it proved difficult to reproduce the experimentally observed peaks between 3200 and 3700 cm^–1^, especially for the high frequency part in the αEtPhCbi^+^ diastereomer but also elsewhere, as it appears that perhaps hydrogen-bonding and anharmonic effects may affect the observed frequencies in that region. We, therefore, turned to an alternative method for assignment of vibrational transitions: BOMD simulations. The resulting BOMD spectra for both the α- and β- diastereomers are shown in Figure 2c and f.

## Discussion

Coenzyme B_12_ (adenosylcobalamin, AdoCbl) is the cofactor for a large number of enzymes in which it functions as a reversible radical source^15^ via homolytic cleavage of the relatively weak Co–C bond and subsequent full reconstitution of the latter.^3,4,38,39^ Remarkably, the same adenosyl radical is also produced from cleavage of the simpler *S*-adenosylmethionine (SAM), induced by a one-electron reduction, initiating similar radical chemistry in enzymes requiring SAM as a cofactor.^40,41^ Hence, it is interesting to learn the means by which the B_12_-cofactors offer advantages beyond SAM and to elucidate the key structural features of the natural cobalt-corrins contributing to their capacity to serve as biologically important organometallic cofactors. The Co–C bond of AdoCbl is inherently weak and furnishes an Ado-radical reversibly by homolysis. In aqueous solution, various experimental determinations of the AdoCbl Co–C bond strength have delivered results close to the reported value of 31.4 kcal mol^–1^.^42−44^ Our recent gas phase measurement of the Co–C bond strength of adenosylcobinamide, the core moiety of AdoCbl lacking only the benzimidazole nucleotide function at the f-side chain, furnished the value of 41 kcal mol^–1^.^45^ Importantly, the critical Co–C bond homolysis of AdoCbl is amenable to very effective activation in AdoCbl-dependent enzymes. Based on a mechanism with one Co–C cleavage per turnover in B_12_-dependent enzymes, from the turnover frequency of the typical AdoCbl-dependent enzymes under physiological conditions an upper bound on the Co–C bond strength of 16 kcal mol^–1^ has been derived.^46^ The observed range of Co–C bond energies, from 41 to 31 to less than 16 kcal mol^–1^ (in the enzyme), is extraordinary as, in general, *homolytic* bond dissociation energies do NOT exhibit strong dependence on the medium. Clearly, a low effective bond strength of below 16 kcal mol^–1^ is a *sine qua non* for the function of coenzyme B_12_ as an enzyme cofactor and the basis of efficient catalysis. The deduced roughly 10^12^ times acceleration of the Co–C bond homolysis in typical AdoCbl-dependent enzymes is a proposed consequence of strain exerted in the protein, once the enzymes are loaded with the substrate molecules.^47^ It is difficult to escape the thought that the imposable variability in the bond strength of the Co–C bond of the AdoCbl B_12_-cofactor is a specific feature of its evolved complex structure. Indeed, acetamide side chains have been observed to help provide a binding interface for the fleetingly existent Ado-radical in the AdoCbl-dependent eliminating isomerases^48^ and glutamate mutase.^47^ These biostructural observations strengthen the wider implications of our recent experimental report that the Co–C bond dissociation energy in the related arylcobinamides depends on the conformational remodeling of the peripheral side chains around the corrin moiety when the central Co–C bond is cleaved.^16^

Early computational studies of vitamin B_12_ and its function truncated the ligand by removing the peripheral side chains to simplify the calculation,^49,50^ which, in light of our recent report, undermined an adequate description of the thermodynamics of the Co–C cleavage. More recent calculations include the side chains, but, the conformational complexity presents an immense challenge.^51^ We reported a combined experimental and computational study of well-defined molecular ions with systematically increased conformational complexity;^52^ the results found that it becomes increasingly difficult for present computational workflows to achieve even the minimum task of identifying the lowest energy structure, once the number of opposing, or compensating, interactions exceeds a certain threshold. Given the experimental observation, *vide supra*, that the Co–C bond dissociation energy in the cobinamides depends sensitively on the network of noncovalent interactions with and among the side chains, we conclude that any even minimally adequate computational model for the function of vitamin B_12_ requires the reliable identification of the lowest energy structure with its network of noncovalent interactions. Given that we have measured the Co–C bond strength in the gas phase, it is correspondingly necessary that we can determine the ground state structure, including the network of noncovalent interactions, also in the gas phase. We suggest that experimental access to and identification of the lowest energy structure can be done by gas-phase vibrational spectroscopy of well-defined cobinamide molecular ions at low temperature. We further propose, as will be seen below, that the number and position of sharp, high-frequency bands, assigned to free (non-H-bonded) N–H and O–H stretches, serve as a fingerprint for the lowest energy conformer of a gas-phase cobinamide. The fingerprint gives us the opportunity to test the adequacy of any given electronic structure method for the prediction of the lowest energy conformation of the side chains, from which one might expect a good chance of an adequate prediction of the Co–C bond strength and its subsequent modulation during enzyme catalysis.

The present “best practices” computational workflow for complicated (bio)molecules in the condensed phase works with an ensemble of conformations found by simulated annealing or a metadynamics search, for which geometry optimizations are done by *ab initio* or, in the case of molecules as large as the cobinamides, DFT methods. For each gas-phase structure within a set energetic range above the global minimum, some solvent model, most commonly a generalized Born (implicit) solvent model, corrects the energies of the molecules from the gas phase into solution. Given the method-based uncertainties, or potential pathologies, in both the electronic structure calculation and the solvent model, one would ideally need independent experimental validation of each of the two “halves” of the composite prediction of the structure/energetics in condensed phase. Concretely, the experimentally measured BDE of AdoCbl or AdoCbi^+^ (the cobalamin and the cobinamide, respectively) in water can be compared to a composite prediction from a DFT calculation and a polarizable continuum method (PCM) solvent model, but adventitious error cancelation in the two calculations could render an agreement to experiment merely fortuitous. As an indication that the composite calculation does indeed suffer from some (unknown) problem, a recent, extensive computational investigation that accounted for solvent effects^51^ calculated a 6.8 kcal/mol weaker bond in methylcobalamin compared to that of methyl-cobinamide, in water. Disappointingly, the calculated stability order does not agree with the experimental observation by Kräutler, in a direct equilibration experiment in water,^53^ that the Co–C bond in methylcobalamin is stronger than the corresponding bond in methylcobinamide. In the composite calculation, there is no way to determine whether the fault lies with the DFT calculation, the solvent model, or both. Our original gas-phase determination^45^ of the BDE in MeCbi^+^ and AdoCbi^+^ provides the additional data needed to validate (or not) both the electronic structure calculation and the solvent model independently, at least for the cobinamides. Similarly, the present spectroscopic study probes the adequacy, or lack thereof, of the electronic structure method for the treatment of the noncovalent interactions that modulate the BDE, in the gas phase, and in solution. The latter requires the additional application of a solvent model, but as we argue above, a credible prediction of condensed-phase behavior by a composite calculation needs both the electronic structure method and the solvent model to be good enough.

CIVP spectroscopy represents a good probe of the structural properties of an ion in the gas phase. Our experimental CIVP spectra exhibit a number of sharp, narrow peaks in the range 3400–3650 cm^–1^. This means that, although the ion structure has been predicted to be compact in some computational studies,^51^ meaning that it is anchored by a rich network of hydrogen bonds, experiment shows that many of the N–H bonds remain nevertheless “free”, *e.g.*, not participating in the hydrogen bonding of the side chains. As we define the experimental metric encoding a conformation in the gas-phase cobinamides, we consider that the H-bonded N–H and O–H bands are not only red-shifted but also (potentially) perturbed. In our previous studies,^54^ and in comparable experiments by other groups,^55−59^ H-bonding lowered the frequency of the affected N–H or O–H band by as much as 1000 cm^–1^. The red-shifted bands are often split or broadened by interaction with one or more “dark” overtones of transitions involving skeletal vibrations, with the upper states of the “bright” transitions involving C–H, N–H, and O–H stretches, whose frequency is approximately double that of the fundamental for the “dark” overtone. As the fundamental frequencies of most of these skeletal modes lie between 1200 and 1500 cm^–1^, the interaction, induced by anharmonicity and conventionally called a Fermi resonance, distributes the oscillator strength of the “bright” fundamentals over a manifold of mixed bands.^60^ In the case of a sparse manifold of states, the typical case of Fermi resonances, the perturbed transition appears as a cluster of bands. For a dense manifold of states, one sees a broadening of the band, which can, in the extreme case, cause the band to “hide in plain sight.”^57^ For the present case, the sharp bands above 3400 cm^–1^ are unlikely to be perturbed, as there are very few, if any at all, skeletal modes at half their frequency. The splitting and broadening accordingly pertain primarily to the red-shifted bands associated with H-bonded chromophores. Consequently, the principal, experimentally observable, and easily interpretable readouts for the conformation comprise the number and position of the sharp, higher-frequency bands.

Given the usual, more-than-adequate, agreement of computed vibrational spectra with experimentally measured IR or Raman spectra,^61−63^ and with the argument above for using the number, position, and identity of the high-frequency, non-H-bonded stretches as the experimental readout encoding conformation, one would expect that the identity of the lowest energy conformation of the cobinamides, in the gas phase, can be confirmed (or not) by direct comparison of the computed IR spectrum with the experimentally measured CIVP spectrum. Figure 2 shows the IR spectra, computed with BP86-D3/def2-TZVP under the harmonic approximation, compared to the CIVP spectra for the α- and β-diastereomers of EtPhCbi^+^, from which the immediate reaction is that the match, especially for αEtPhCbi^+^, is poor. While anharmonicity in the high-frequency stretches is not taken into account, the non-H-bonded N–H and O–H stretches should be among the normal modes least affected by anharmonicity, anyways. In our experience, harmonic frequencies, computed with BP86-D3 and a triple-ζ basis set, for C–H, N–H, and O–H stretches need scaling by factors very close to unity, *e.g.*, 0.98 or 0.99 to match experiment.^64,18,52^ This latter claim can be further supported by the general similarity between the harmonic spectra and the experimental IR spectra for many small molecules and ions, especially in the high-frequency part of the spectrum.

**Figure 3:**
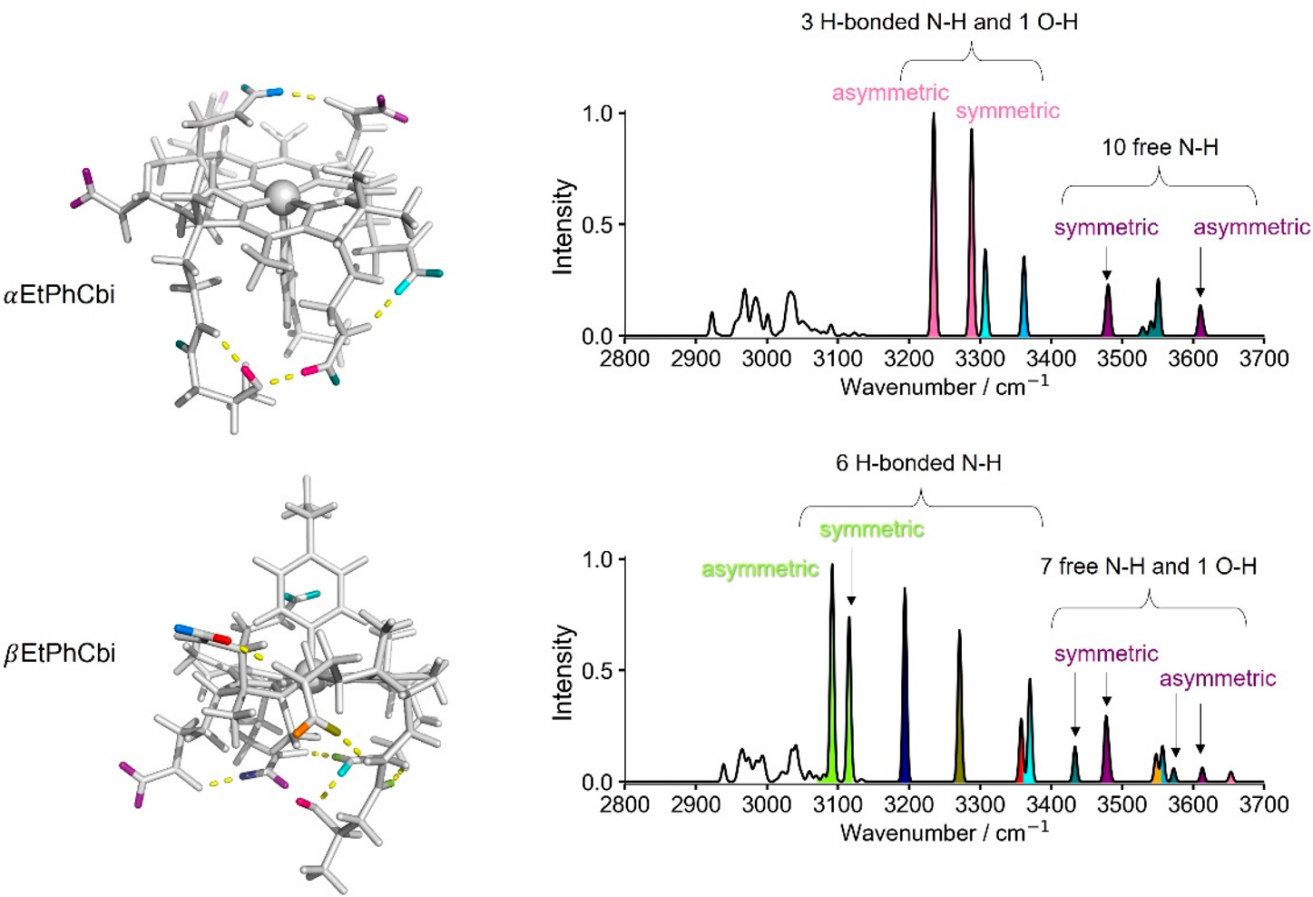
Calculated structures and corresponding harmonic IR spectra using the BP86-D3 functional and def2-TZVP basis set. Individual N–H and O–H bonds as well as the observed stretching frequencies are color-coded.

If we consider the sharp bands above 3400 cm^–1^ in the experimental CIVP spectra, Figure 1, we count 10 for αEtPhCbi^+^ and 7 or 8 for βEtPhCbi^+^, out of the potential 13 N–H and 1 O–H stretches. Just from the visual count, the numbers could be taken as ±1, given some overlap between bands as well as what appears to be a very low intensity peak just discernible above the noise slightly above 3600 cm^–1^ for αEtPhCbi^+^, which may or may not be a *bona fide* band, and what appears to be a very close pair of bands at 3400 cm^–1^ in βEtPhCbi^+^. A direct comparison of the *number* of sharp bands above 3400 cm^–1^ to the predictions based on the harmonic spectrum calculated with BP86-D3/def2-TZVP proves quite satisfactory, with the computed spectrum showing 10 for αEtPhCbi^+^ and 7 for βEtPhCbi^+^, based on the correlation of normal frequencies to normal mode atom displacements. Figure 3 shows the computed harmonic spectra, with the color-coded bands marked according to whether the particular N–H or the O–H bond is H-bonded, or not, in the computed minimum energy structure. Note that calculation confirms our cutoff of 3400 cm^–1^ as an indicator of H-bonded status. Note further that, for αEtPhCbi^+^, the 10 bands corresponding to free N–H and O–H functionalities are bunched together in the experimental spectrum with enough overlap to make the count a bit difficult. The computation considers neither anharmonicity nor perturbations, so, while the red-shift of bands associated with H-bonded moieties appears nicely, any splitting or broadening of the bands remains untreated at the harmonic level of analysis.

While the *number* of bands assigned to non-H-bonded N–H and O–H moieties appears satisfactory in the BP86-D3/def2-TZVP structure, the *positions* and the *intensities* of the bands above 3400 cm^–1^ are reproduced shockingly poorly. While one may find the latter of lesser concern, the large deviation of computed from observed frequencies is truly disappointing. As argued above, theory and prior precedence would lead us to expect much better agreement. Particularly striking, if only because they stand out in the spectra, are the very intense, highest frequency bands at 3639 and 3620 cm^–1^ in αEtPhCbi^+^ and βEtPhCbi^+^, respectively, in a part of the spectrum where BP86-D3/def2-TZVP predicts that there should be either nothing large or nothing at all.

Seeking to investigate whether the disappointing degree of agreement between the computed and observed vibrational spectra may be due to the use of the harmonic approximation in the former, we extracted the simulated IR spectra from Born–Oppenheimer molecular dynamics (BOMD) trajectories. The method, termed FT-DAC, uses the Fourier transform of the dipole autocorrelation function to extract the theoretical vibrational spectrum from MD trajectories done with any given electronic structure method. While our earlier reports used the fast, semiempirical xTB approximation to DFT,^65^ we executed the trajectories used in Figure 2 fully numerically with BP86-D3, albeit with a double-ζ basis set, for “on the fly” calculation of the energy and gradient at each time step. We further checked trajectories, initiated with different initial conditions but starting at the previously minimum energy structure, and let them run for 20 ps. The design of the BOMD study should provide a reasonable treatment of any anharmonicity of the potential function in the vicinity of the computed minimum, in general, and it should allow for sampling of adjacent minima if the barriers were to be very low, which is a case of which we were concerned. Looking at the region above 3400 cm^–1^ in Figure 2, one sees a gratifyingly good agreement between the FT-DAC-computed spectra and the corresponding harmonic spectra when the same electronic structure method is used for both, which suggests that our presupposition that even the straightforward, harmonic approximation should be appropriate for the high-frequency bands is likely to be correct. This latter conclusion does not, however, resolve the disagreement between the computed versus experimental spectra.

Other studies in the literature implemented VPT2, a second-order perturbation theory approach, or direct diagonalization of a truncated secular matrix, to treat anharmonicity and perturbations, including Fermi resonances.^66^ We had tried the former method, which unsurprisingly gave disappointing results for strongly perturbed bands beyond the scope of effects due to “small” interactions. In the case of the nominally unperturbed bands above 3400 cm^–1^, significant differences were neither expected nor found. Treatment of strongly perturbed bands by direct diagonalization has been successful for Fermi resonances in the spectra of small molecules and molecular ions,^67,68,60^ but this treatment could not be implemented for the present case of a very large molecular ion, where the sheer number of interactions, combined with ambiguities in extracting off-diagonal matrix elements, stymied an effort which would have been unlikely to produce any large change in the target high-frequency bands.

**Figure 4:**
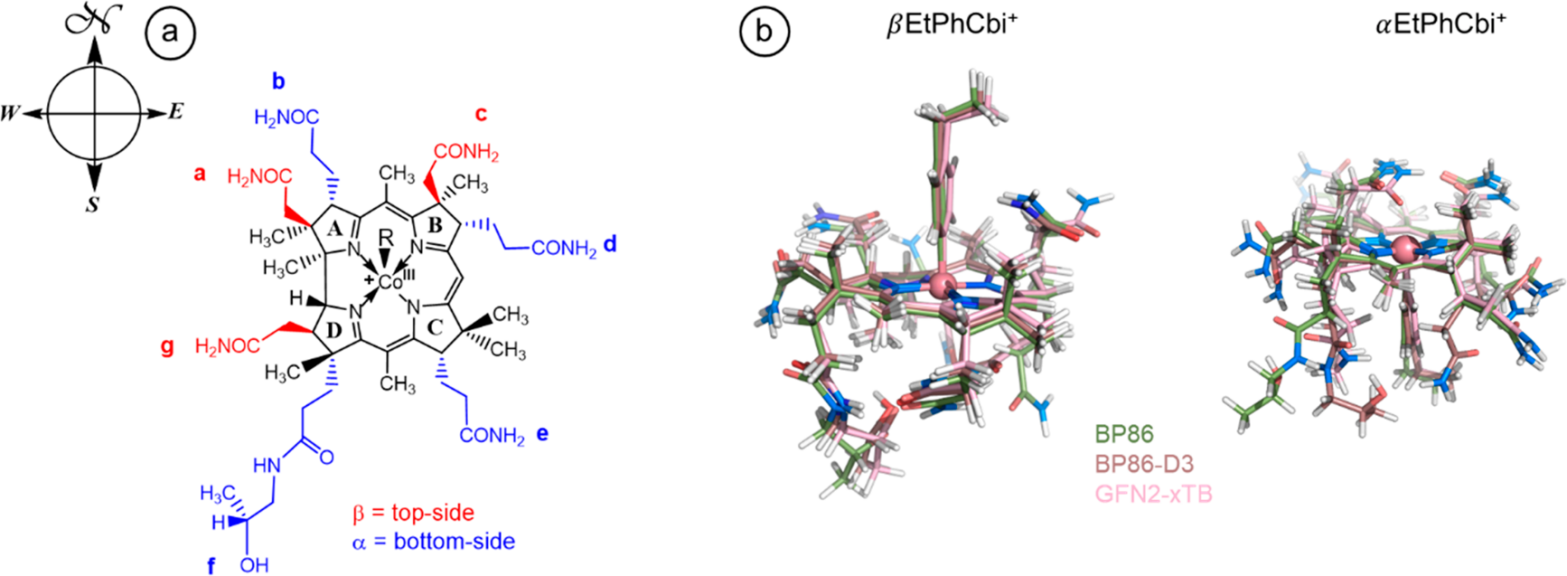
(a) Conventional designation of rings, side-chains, and orientation in cobinamides; (b) overlay of the minimum-energy structures calculated with different methods for βEtPhCbi^+^ and αEtPhCbi^+^.

Having previously found that an increasing number of noncovalent interactions acting against each other confounds our ability to predict the minimum energy conformation reliably for medium-sized to large molecules, it is reasonable to consider whether the predicted minimum energy conformations for αEtPhCbi^+^ and βEtPhCbi^+^ are robust with regard to a change in the treatment of those noncovalent interactions. A crude, if still effective, test comes from the search for the minimum energy structures for αEtPhCbi^+^ and βEtPhCbi^+^ with BP86/def2-TZVP, in which the removal of the D3 correction reduces the long-range attractive part of the van der Waals potential. Many benchmark studies find that the typical DFT functionals, BP86 among them, systematically underestimate attractive forces in the 3–5 Å range.^25,26,69−71^ Recent work on a variety of test systems using multiple different experimental techniques suggests strongly that the D3 correction overestimates that attractive interaction, at the very least for certain interaction geometries.^72^ However, one may judge the evidence, it is probably fair to claim that the physically most realistic treatment for London dispersion interactions lies somewhere between no D3 and 100% D3, most likely toward the higher end but not necessarily uniformly for all interactions at all geometries. Taking this situation as a starting point, one may test for robustness in the optimized structure, *i.e.* the predicted lowest energy conformation, of αEtPhCbi^+^ and βEtPhCbi^+^ by comparing the network of H-bonded peripheral side chains with and without the D3 correction. If the network of H-bonds were to remain invariant, then one could claim that the present computational workflow would have a reasonable probability of identifying the correct conformation, with the correct network of noncovalent interactions, from which one would begin to explain the modulation of the Co–C bond dissociation energy in vitamin B_12_. Figure 4 shows the conventional designation of the side-chains in cobinamides and cobalamines. Because the network of interactions is difficult to read directly from the overlaid structures in Figure 4, Tables 1 and 2 depict the pattern of H-bonding for BP86, BP86-D3, and GFN2-xTB (used in the early stages of geometry optimization). Evident from Tables 1 and 2 is the clear qualitative difference between the H-bonding network with and without the D3 correction. While a definite conclusion would require a finer-grained scaling of the noncovalent interactions, it appears that adding or withholding the D3 correction leads the molecule to switch between two different structural motifs. The overlaid structures do not appear overly different, but under the apparent similarity due to the common corrin core, the side chains look like they can switch partners so that the pattern of H-bonds changes greatly even if the number of H-bonds does not. The scope of the problem is visible during the geometry optimization process. As we had previously reported, the CREST conformer search at the onset of the optimization found approximately 300 conformers within 6 kcal mol^–1^ of the minimum, and we chose to reoptimize the ten best structures whose CREST energies spanned 2 kcal mol^–1^. Even these structures showed diversity in their network of interactions, making it certainly plausible that DFT finds the wrong structure in its search for the lowest energy conformation.

**Table 1:**
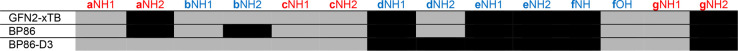
Side-Chain Map of the Free (Black) Versus Hydrogen-Bonded (Gray) Side Chains in βEtPhCbi^+^ for the Most Stable Structures Optimized with Different Methods

**Table 2:**
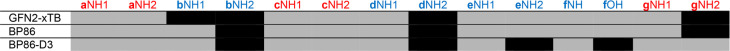
Side-Chain Map of the Free (Gray) Versus Hydrogen-Bonded (Black) Side Chains in αEtPhCbi^+^ for the Most Stable Structures Optimized with Different Methods

Good scientific practice demands that we consider that the experiment may have some essential flaw. While the CIVP spectra, especially for the very large molecular ions in this study, can be justifiably treated as if they were IR spectra, and the low laser power means that we have a “linear” spectroscopy with no significant power broadening or other artifacts, one can nevertheless ask two questions: whether the molecular ions are, in fact, equilibrated to their lowest energy structures and whether the so-called “tag effect” can have any influence on the structure.

First, we would like to address the question of whether there is a plausible chance that the ions have been trapped in some local minimum, *i.e.*, a metastable structure, during the cooling process. Our CIVP spectrometer was constructed around a cryogenic Penning trap or, equivalently, a low-temperature FT-MS. The ions are produced by electrospray from solution and transmitted to an ion funnel—a radio frequency ion guide that effectively collects and concentrates the divergent stream of ions exiting the electrospray capillary— prior to mass-selection by a quadrupole mass filter. The ions should not be overly “warm” before they are conducted into the Penning trap. The cooling of the ions is achieved in the trap itself, with the background gas, 10^–8^ mbar, thermalized to between 10 and 50 K, as set by a closed-cycle cryostat. Slow collisional cooling means that the ions should not be caught in metastable states. Time scales for holding the ions in the cryogenic Penning trap, before and during irradiation, are on the order of minutes, during which there are collisions with the cold background gas, again mitigating against nonequilibrium distributions over states accessible at the low temperature. We conclude that it is implausible that the ions in this study do not have the lowest energy structure.

Second, the issue of the tag effects demands additional discussion. Because CIVP spectroscopy requires the use of weakly bound messenger tag molecules such as N_2_, which bind to the ion of interest at low temperatures, and dissociate when the ion absorbs an IR photon, one needs to consider the possibility of structural perturbation, influenced by this binding. In our earlier studies, we thoroughly explored the impact of the H_2_ and N_2_ binding on various molecular structures and systems.^18,65^ We found that the H_2_ or N_2_ tag tends to perturb the spectra of the corresponding ion significantly only under specific circumstances, typically arising when the tag binding goes beyond simple ion-induced dipole interactions, *i.e.*, when the tag coordinates as a ligand, or when there is incipient H-bonding. In such cases, an NH or OH band can shift or split, or, more dramatically, the otherwise dipole-forbidden vibrational mode of the tag itself becomes “IR active.” Specifically considering the case of the cobinamides, a unique scenario could arise if the N_2_ moiety were to be bound as a ligand to the cobalt site. However, we note that we do not see the characteristic stretching frequency associated with N_2_ in our experimental spectra. Furthermore, separate spectra for clusters with one, two, or three N_2_ tags (Figures S10 and S11 in the SI) differ from each other, and from the composite (Figure 1), no more than between spectra taken on different days, especially in the region >3400 cm^–1^, suggesting that the tags are not interacting specifically with the chromophores we designate as the structural descriptors. These observations lead us to conclude that there is no strong perturbation induced by the N_2_ tag in our current study.

Returning to the original issue that motivated a structure-sensitive spectroscopic study of the arylcobinamides in the gas phase, the comparison of the number and position of peaks above 3400 cm^–1^ to predictions by DFT, which had been expected to give at least qualitative agreement, finds discrepancies that we believe indicate that the applied methods failed to find the minimum energy structure, *i.e.*, the lowest energy conformation, reliably for a molecule with the size and flexibility of an aryl cobinamide. The disappointing result is consistent with observations made in our previous work on the conformations of ions used as molecular torsion balances, in which multiple, weak, noncovalent interactions were balanced against each other. Perhaps it is not surprising that a minimally physically realistic treatment of complex conformational equilibria poses exceptional challenges for computational methodology, given that the final compromise depends on highly accurate calculation of many, many weak forces, sometimes reinforcing and sometimes canceling each other out. According to the hypothesis that the conformation of the peripheral side chains modulates the Co–C bond dissociation energy in vitamin B_12_, we believe that the presently practical computational methods are not yet adequate to treat this extraordinarily complicated, but also extraordinarily important, case.

## Conclusion

Vibrational spectroscopy should function as a probe of the gas-phase structure or, more specifically, the conformation of ions of biomolecular importance, and one would hope that harmonic analysis, or, at one level higher, BOMD analysis, can render the spectroscopic results physically interpretable for ions as big as cobinamides. The goal is a better understanding of the structure–function relationship in large biomolecules. Having obtained clean, well-resolved CIVP spectra of α- and β-aryl cobinamides, we nevertheless find that the presently practical computational workflows do not provide convincing predictions of the vibrational spectra, which after we exclude confounding factors in the spectra, we interpret to mean that the applied methods failed to identify the lowest energy conformation in the cases of large, meaning ca. 150 atoms, flexible molecular ions. More accurate theoretical methods are needed to test the hypothesis. Being unable to solve the gas-phase structures of the aryl cobinamides investigated here convincingly with the available computational approaches, we aim to assign the experimentally observed vibrational modes by an isotopic labeling approach. Briefly, the ongoing work includes synthesis of different specifically monolabeled B_12_ analogs by hydrolysis of single amide-groups and their subsequent amidation with ^15^NH_3_. CIVP spectra of these derivatives will allow us to resolve unambiguously which side chains participate in hydrogen bonding and which remain free in the structures that we measure. An experimentally unambiguous correlation of observed bands to the corresponding structural unit on the cobinamide should provide a hard benchmark for any further development of computational methods and conformational searches.

## Supporting Information Available

General information about materials and methods; further computational results (PDF)
xyz files of all optimized structures as well as ASCII xy files of the experimental CIVP spectra (ZIP)
