# Supplementary material for: Cryogenic Ion Vibrational Predissociation (CIVP) Spectroscopy of Aryl Cobinamides in the Gas Phase: How Good Are the Calculations for Vitamin B12 Derivatives?
Source: J Am Chem Soc. 2023 Sep 1;145(36):19561–70. doi: 10.1021/jacs.3c03001 (PMC10510309; doi:10.1021/jacs.3c03001)
Supplement: Supplementary file 1 — ja3c03001_si_001.pdf [file ja3c03001_si_001.pdf]

Supporting Information

Cryogenic ion vibrational predissociation (CIVP) spectroscopy of aryl cobinamides in the gas phase: how good are the calculations for vitamin B_{12} derivatives?

Alexandra Tsybizova, Lukas Fritsche, Larisa Miloglyadova, Bernhard Kräutler and Peter Chen*

Laboratorium für Organische Chemie, ETH Zürich, Vladimir-Prelog-Weg 2, 8093 Zürich, Switzerland

*E-mail: peter.chen@org.chem.ethz.ch

*phone: +41 44 632 28 98

Contents

S1 Abbreviation list **S5**

S2 Computational analysis **S6**

S2.1 Harmonic DFT calculations S6
S2.2 Harmonic GFN2-xTB calculations S13
S2.3 Semiemperical BOMD simulations S13
S2.4 CP2K calculations S18

S3 Experimental evaluation of tag effect **S24**

References **S26**

List of Figures

S1 Comparison of the experimental CIVP spectra of a) β- and b) α-4-EtPhCbi with the calculated harmonic IR spectra using BP86 functional and def2-TZVP basis set, scaling factor used: 0.99. . . . . S11

S2 Comparison of the experimental CIVP spectra of β- and α-4-EtPhCbi with the calculated harmonic IR spectra for the respective ten and nine lowest-energy conformers from the CREST search, using BP86-D3 functional and def2-TZVP basis set. Conformer 10 is omitted for α-4-EtPhCbi due to the problems with SCF convergence during DFT optimization. Scaling factor used: 0.98. . . . . S12

S3 Comparison of the experimental CIVP spectra of a) β- and b) α-4-EtPhCbi with the calculated harmonic IR spectra using semiemperical GFN2-xTB method. S13

S4 Experimental CIVP spectrum of a) β- and b) α-4-ethylphenylcobinamide together with the FT-DAC-based GFN2-xTB spectra. S14

S5 Spectra calculated via FT-DAC averaged over fifty MD trajectories for α-4-EtPhCbi at 50 K, together with spectra extracted for individual free N-H and O-H bonds (left) as well as hydrogen-bound N-H bonds (right). S16

S6 Spectra calculated via FT-DAC averaged over fifty MD trajectories for βEtPhCbi at 50 K, together with spectra extracted for individual free N-H and O-H bonds.S17

S7 Spectra calculated via FT-DAC averaged over fifty MD trajectories for βEtPhCbi at 50 K, together with spectra extracted for individual hydrogen-bound N-H bonds. S18

S8 Comparison of the experimental CIVP spectra of a) β- and b) α-4-EtPhCbi with the calculated BOMD IR spectra using BP86/MOLOPT-DZVP-SR-GTH method. S22

S9 Comparison of the experimental CIVP spectra of a) β- and b) α-4-EtPhCbi with the calculated harmonic IR spectra using BP86-D3/MOLOPT-DZVP-SR-GTH method, as well as the BOMD spectra produced with the same method. S23

S10 Comparison of the experimental CIVP spectra α-4-EtPhCbi recorded with different number of N_2 tags. S24

S11 Comparison of the experimental CIVP spectra β-4-EtPhCbi recorded with different number of N_2 tags. S25

List of Tables

S1 Abbreviations used in the text S5

S2 Calculated high-wavenumber stretching frequencies for the most stable structure of βEtPhCbi$^+$, obtained with BP83-D3/def2-TZVP level of theory. All frequencies are scaled by 0.99 to match the observed C-H stretching region. The labeling follows the conventional designation of the side chains, shown in the main text, Figure 4. . . . . S7

S3 Calculated high-wavenumber stretching frequencies for the most stable structure αEtPhCbi$^+$, obtained with BP83-D3/def2-TZVP level of theory. All frequencies are scaled by 0.99 to match the observed C-H stretching region. The labeling follows the conventional designation of the side chains, shown in the main text, Figure 4. . . . . S8

S4 Calculated high-wavenumber stretching frequencies for the most stable structure βEtPhCbi$^+$, obtained with BP83/def2-TZVP level of theory. All frequencies are scaled by 0.99 to match the observed C-H stretching region. The labeling follows the conventional designation of the side chains, shown in the main text, Figure 4. . . . . S9

S5 Calculated high-wavenumber stretching frequencies for the most stable structure αEtPhCbi$^+$, obtained with BP83/def2-TZVP level of theory. All frequencies are scaled by 0.99 to match the observed C-H stretching region. The labeling follows the conventional designation of the side chains, shown in the main text, Figure 4. . . . . S10

S1 Abbreviation list

Table S1: Abbreviations used in the text

CIVP	Cryogenic ion vibrational predissociation
BOMD	Born-Oppenheimer molecular dynamics
ICR	Ion cyclotron resonance
DFT	Density functional theory
ESI	Electrospray ionization

S2 Computational analysis

S2.1 Harmonic DFT calculations

As was mentioned in the Results section, DFT calculations were performed with Gaussian 09 suite (revision D.01).[1]

To allow better comparison with the experiment, Tables S2-S4 summarize calculated high-wavenumber stretching frequencies (scaled by 0.99 to match the observed C-H stretching frequencies) for the most stable structure of βEtPhCbi$^+$ and αEtPhCbi$^+$, calculated either at BP83-D3/def2-TZVP or BP83/def2-TZVP level of theory.

Table S2: Calculated high-wavenumber stretching frequencies for the most stable structure of βEtPhCbi$^+$, obtained with BP83-D3/def2-TZVP level of theory. All frequencies are scaled by 0.99 to match the observed C-H stretching region. The labeling follows the conventional designation of the side chains, shown in the main text, Figure 4.

compound	wavenumber, cm^{-1}	IR intensity	side chain and bond label	type of stretch
βEtPhCbi$^+$	3054.76	3.08	C-H	
	3060.86	604.14	e-NH-perturbed + e-NH perturbed + f-NH	asymmetric
	3069.45	1.65	C-H	
	3084.44	460.57	e-NH-perturbed + e-NH perturbed + f-NH	asymmetric
	3088.88	3.94	C-H	
	3101.78	6.66	C-H	
	3108.75	0.23	C-H	
	3162.18	536.78	d-NH free + d-NH-perturbed	symmetric
	3238.46	419.63	g-NH-free + g-NH-perturbed	symmetric
	3324.22	174.53	a-NH-free + a-NH-perturbed	symmetric
	3336.39	284.88	e-NH-perturbed + e-NH-perturbed	asymmetric
	3399.16	98.08	c-NH-free + c-NH-free	symmetric
	3442.09	164.96	d-NH-free + d-NH-perturbed	asymmetric
	3446.22	61.88	b-NH-free + b-NH-free	symmetric
	3512.11	77.09	g-NH-free + g-NH-perturbed	asymmetric
	3521.07	99.44	a-NH-free + a-NH-perturbed	asymmetric
	3536.59	37.30	c-NH-free + c-NH-free	asymmetric
	3576.54	39.10	b-NH-free + b-NH-free	asymmetric
	3616.34	27.56	f-OH free	

Table S3: Calculated high-wavenumber stretching frequencies for the most stable structure αEtPhCbi$^+$, obtained with BP83-D3/def2-TZVP level of theory. All frequencies are scaled by 0.99 to match the observed C-H stretching region. The labeling follows the conventional designation of the side chains, shown in the main text, Figure 4.

compound	wavenumber, cm^{-1}	IR intensity	side chain and bond label	type of stretch
αEtPhCbi$^+$	3057.85	13.36	C-H	
	3060.31	18.06	C-H	
	3076.82	4.83	C-H	
	3091.85	8.04	C-H	
	3103.95	3.06	C-H	
	3202.38	592.79	f-OH perturbed + e-NH-petrutbed	asymmetric
	3255.20	546.87	f-OH perturbed + e-NH-petrutbed	symmetric
	3274.25	230.08	d-NH free + d-NH perturbed	symmetric
	3328.02	211.22	a-NH free + a-NH perturbed	symmetric
	3442.97	67.04	b-NH-free + b-NH-free	symmetric
	3446.14	65.51	g-NH free + g-NH-free	symmetric
	3447.06	37.26	c-NH-free + c-NH-free	symmetric
	3493.49	23.17	f-NH-free	
	3505.01	38.50	e-NH-perturbed + e-NH free	asymmetric
	3514.87	66.13	d-NH-free + d-NH-perturbed,	asymmetric
	3515.30	86.07	a-NH free + a-NH perturbed	asymmetric
	3572.93	34.46	g-NH-free + g-NH-free	asymmetric
	3573.41	36.53	b-NH-free + b-NH-free	asymmetric
	3577.05	27.53	c-NH-free + c-NH-free	asymmetric

Table S4: Calculated high-wavenumber stretching frequencies for the most stable structure βEtPhCbi$^+$, obtained with BP83/def2-TZVP level of theory. All frequencies are scaled by 0.99 to match the observed C-H stretching region. The labeling follows the conventional designation of the side chains, shown in the main text, Figure 4.

compound	wavenumber, cm^{-1}	IR intensity	side chain and bond label	type of stretch
βEtPhCbi$^+$	3077.94	5.21	C-H	
	3098.38	0.21	C-H	
	3176.73	482.65	f-NH-perturbed	
	3218.96	443.71	g-NH-free + g-NH-perturbed	symmetric
	3249.71	275.73	e-NH-perturbed + e-NH-perturbed	symmetric
	3293.47	248.57	a-NH-free + a-NH-perturbed	symmetric
	3362.11	488.33	e-NH-perturbed + e-NH-perturbed	asymmetric
	3367.50	118.31	b-NH-free + b-NH free	symmetric
	3408.77	70.09	c-NH-free + c-NH-free	symmetric
	3445.32	61.44	d-NH-free + d-NH free	symmetric
	3504.99	70.91	g-NH-free + g-NH-perturbed	asymmetric
	3509.32	100.90	a-NH-free + a-NH-perturbed	asymmetric
	3515.71	88.26	b-NH-free + b-NH-free	asymmetric
	3535.97	42.54	c-NH-free + c-NH-free	asymmetric
	3573.66	35.16	d-NH-free + d-NH-free	asymmetric
	3624.32	10.28	f-OH-free	

Table S5: Calculated high-wavenumber stretching frequencies for the most stable structure αEtPhCbi$^+$, obtained with BP83/def2-TZVP level of theory. All frequencies are scaled by 0.99 to match the observed C-H stretching region. The labeling follows the conventional designation of the side chains, shown in the main text, Figure 4.

compound	wavenumber, cm^{-1}	IR intensity	side chain and bond label	type of stretch
αEtPhCbi$^+$	3073.84	6.33	C-H	
	3076.36	5.35	C-H	
	3080.89	12.69	C-H	
	3104.65	0.21	C-H	
	3258.85	455.18	g-NH-free + g-NH-perturbed	symmetric
	3370.68	138.13	a-NH-free + a-NH-perturbed	symmetric
	3386.33	153.12	d-NH-free + d-NH-free	symmetric
	3443.63	52.58	c-NH-free + c-NH-free	symmetric
	3444.11	63.30	b-NH-free + b-NH-free	symmetric
	3446.17	59.54	e-NH-free + e-NH-free	symmetric
	3488.56	20.83	f-NH-free	
	3494.96	63.17	g-NH free + g-NH-perturbed	asymmetric
	3530.08	76.19	a-NH-free + a-NH-perturbed	asymmetric
	3534.87	79.54	d-NH-free + d-NH-free	asymmetric
	3573.18	29.86	c-NH-free + c-NH-free	asymmetric
	3574.54	36.05	e-NH-free + e-NH-free	asymmetric
	3574.73	35.42	b-NH-free + b-NH-free	asymmetric
	3576.60	65.42	f-OH-free	

Figure S1 shows the comparison of the experimental CIVP spectra and the harmonic BP86/def2-TZVP spectra, calculated without the dispersion correction.

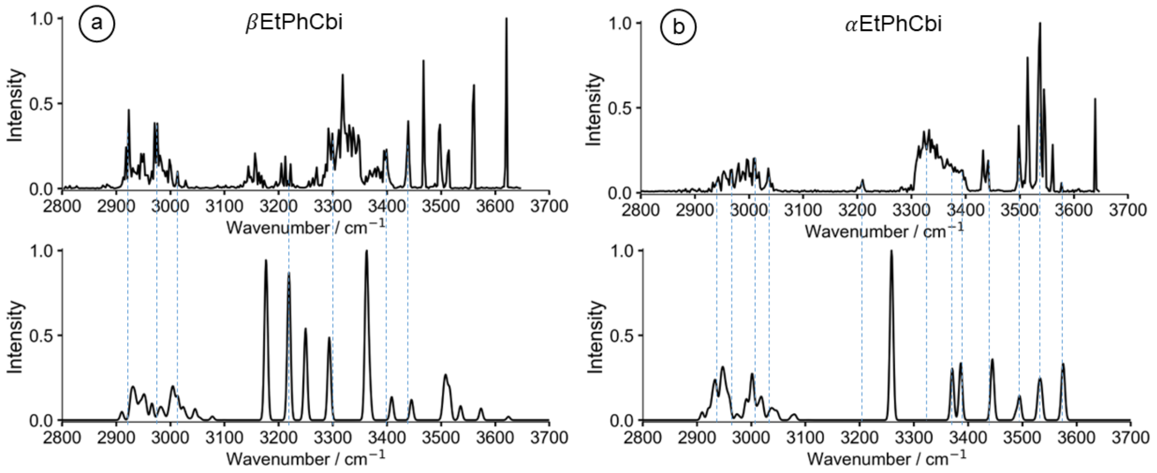

Figure S1: Comparison of the experimental CIVP spectra of a) β- and b) α-4-EtPhCbi with the calculated harmonic IR spectra using BP86 functional and def2-TZVP basis set, scaling factor used: 0.99.

Figure S2 shows the comparison of the experimental CIVP spectra and all the harmonic BP86-D3/def2-TZVP spectra, calculated for all of the reoptimized CREST structures.

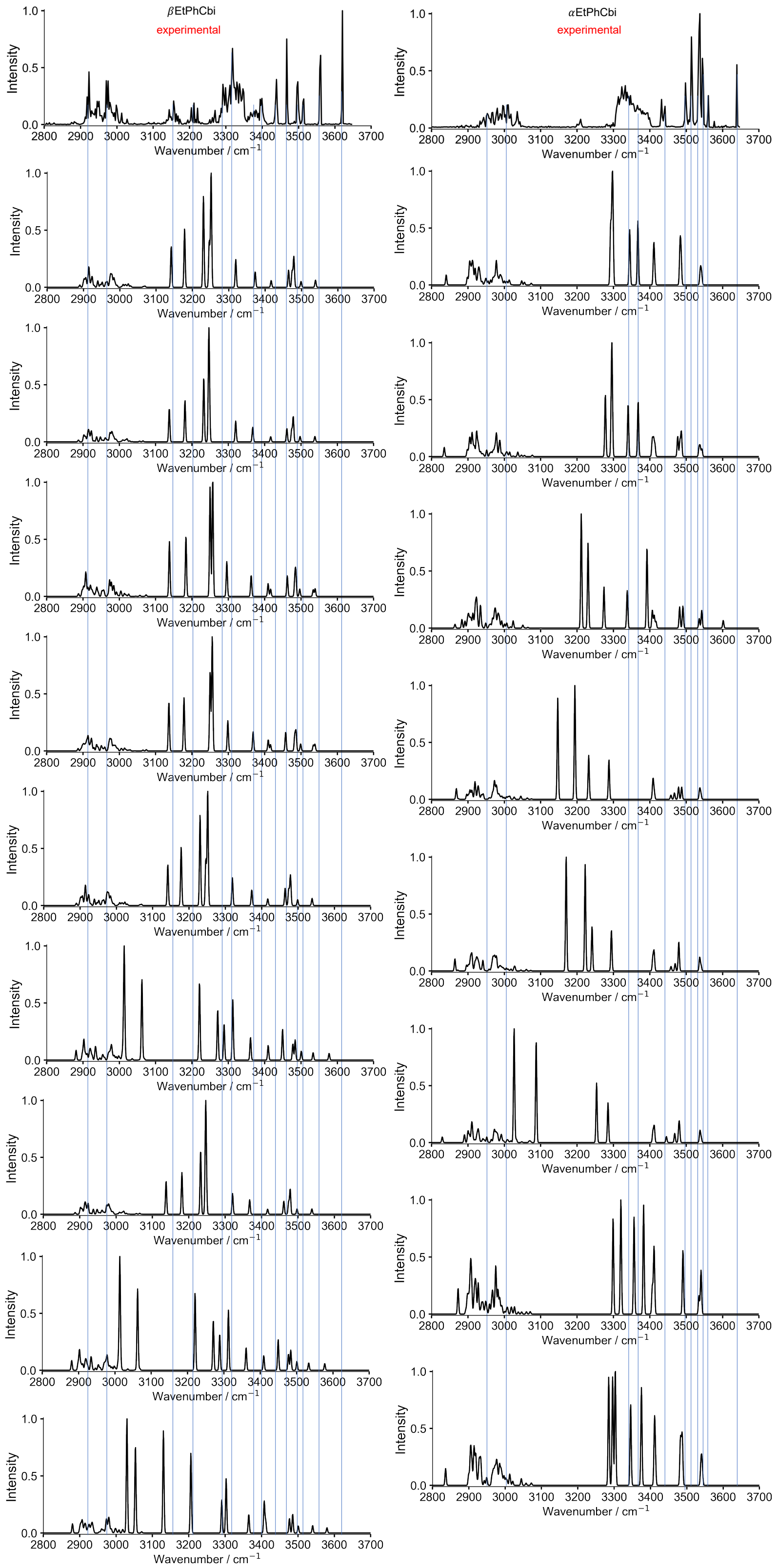

Figure S2: Comparison of the experimental CIVP spectra of β- and α-4-EtPhCbi with the calculated harmonic IR spectra for the respective ten and nine lowest-energy conformers from the CREST search, using BP86-D3 functional and def2-TZVP basis set. Conformer 10 is omitted for α-4-EtPhCbi due to the problems with SCF convergence during DFT optimization. Scaling factor used: 0.98.

S2.2 Harmonic GFN2-xTB calculations

Figure S3 shows the comparison of the experimental CIVP spectra with the harmonic IR spectra calculated with semiemperical GFN2-xTB method.

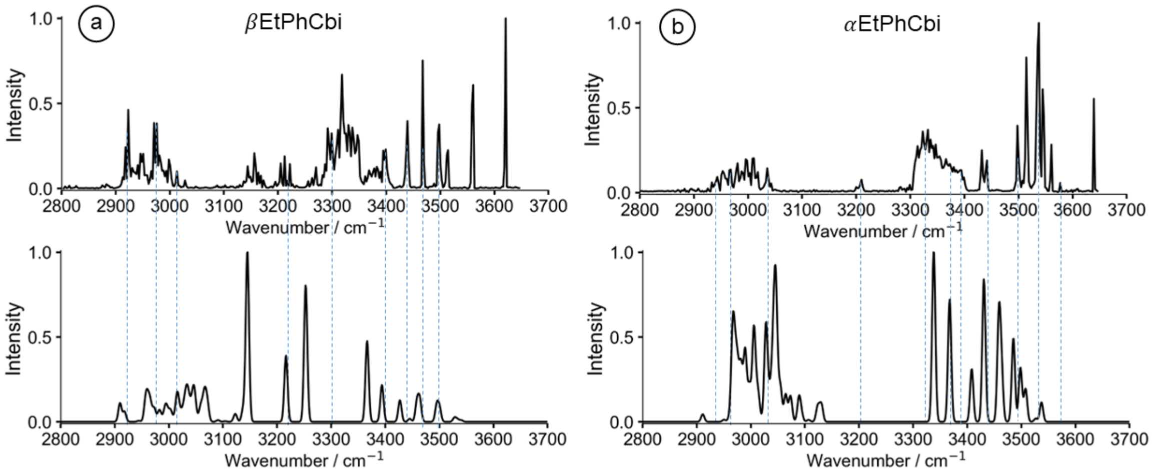

Figure S3: Comparison of the experimental CIVP spectra of a) β- and b) α-4-EtPhCbi with the calculated harmonic IR spectra using semiemperical GFN2-xTB method.

S2.3 Semiemperical BOMD simulations

For the initial analysis, semiemperical BOMD simulations were performed with Atomic Simulation Environment (ASE)[2] 3.18.1 using the GFN2-xTB method[3] via the Python API implemented in xTB 6.2.2. Each calculation started from the best structure identified with the CREST conformational search with a 5 ps NVT simulation using Langevin dynamics, with a friction coefficient of 0.02 a.u., at the target temperature for equilibrating the atomic positions and velocities. The final structure from the NVT simulation was used as the starting point for a 20 ps NVE production simulation. The temperature for the BOMD simulations was set to 50 K to better sample the nuclear motion of interest, and to crudely account for ZPE, similarly to previous work.[4–6] A timestep of 0.5 fs was used in the simulations. In total, 50 trajectories were run for each structure. For calculating the IR spectra from the MD trajectories, the dipole moment vector was extracted at each frame during the simulation. The autocorrelation function of the velocity of the dipole moment was calculated from this data, and the Blackman-Harris window was applied. The IR spectrum was obtained by using discrete fast Fourier transform on the resulting data.[7] We will further refer to this method as FT-DAC. The Python codes used for running the simulations are accessible at GitLab.[8]

Figure S4 shows that the resulting FT-DAC spectra reproduce the broadened peaks that belong to the hydrogen-bonded N-H bonds, as well as some of the sharper peaks in the free N-H and O-H region.

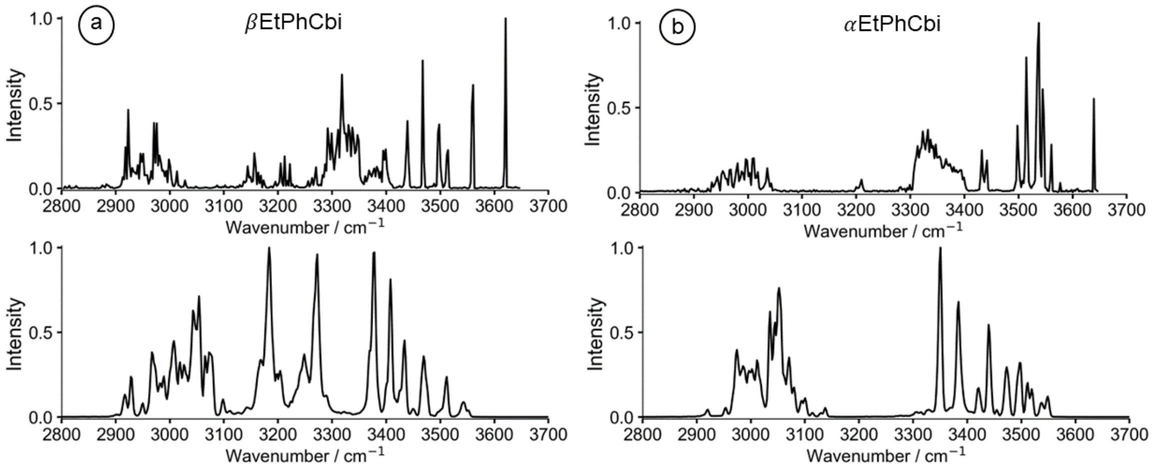

Figure S4: Experimental CIVP spectrum of a) β- and b) α-4-ethylphenylcobinamide together with the FT-DAC-based GFN2-xTB spectra.

To assign which peaks on our simulated spectra belong to the stretching frequencies of which specific bonds, we extracted the dipole moment vector for an isolated bond, calculated the autocorrelation function of the velocity of the dipole moment, and applied the discrete fast Fourier transform on the resulting data. This is necessary because the FT-DAC approach drops the harmonic approximation, which means that the normal mode analysis cannot be performed to assign peaks to specific atomic motions. The individual N-H and O-H bonds for the αEtPhCbi$^+$ structure are depicted in Figure S5, left. The specific bonds for which this analysis was done, as well as the resulting FT-DAC signals, are color-coded. Analogous spectra for the βEtPhCbi$^+$ can be found in Figures S6-S7. As was expected, we confirm that N-H bonds that participate in hydrogen bonding generally gave broader, more red-shifted signals compared to the "free" N-H and O-H bonds. Interestingly, for both αEtPhCbi$^+$ and βEtPhCbi$^+$ the most blue-shifted signal corresponds to the N-H bond on the C-side chain, and not to the free OH bond, as would have been expected based on the dipole moments. The most red-shifted sharp peaks appear to be attributed to "buried" N-H bonds. While these bonds do not participate in the hydrogen bonding of the side chains, but are nevertheless buried inside the framework of the cobinamide ion. The same analysis of the hydrogen-bonded networks in the αEtPhCbi$^+$ structure is shown in Figure S5, right. THe data show how other peaks in the resulting BOMD spectrum can be attributed to each

N-H bond that participates in the non-covalent interactions between the side chains of the ion. While the quantitative agreement of the number of high-frequency versus red-shifted peaks, and their positions, is poor, we believe that the general claim from the simulation, that the red-shifted peaks correspond to H-bonded functions or buried bonds, is legitimate. Accordingly, we can read from the spectra, whether experimental or simulated, the number of free NH or OH bond for any given structure.

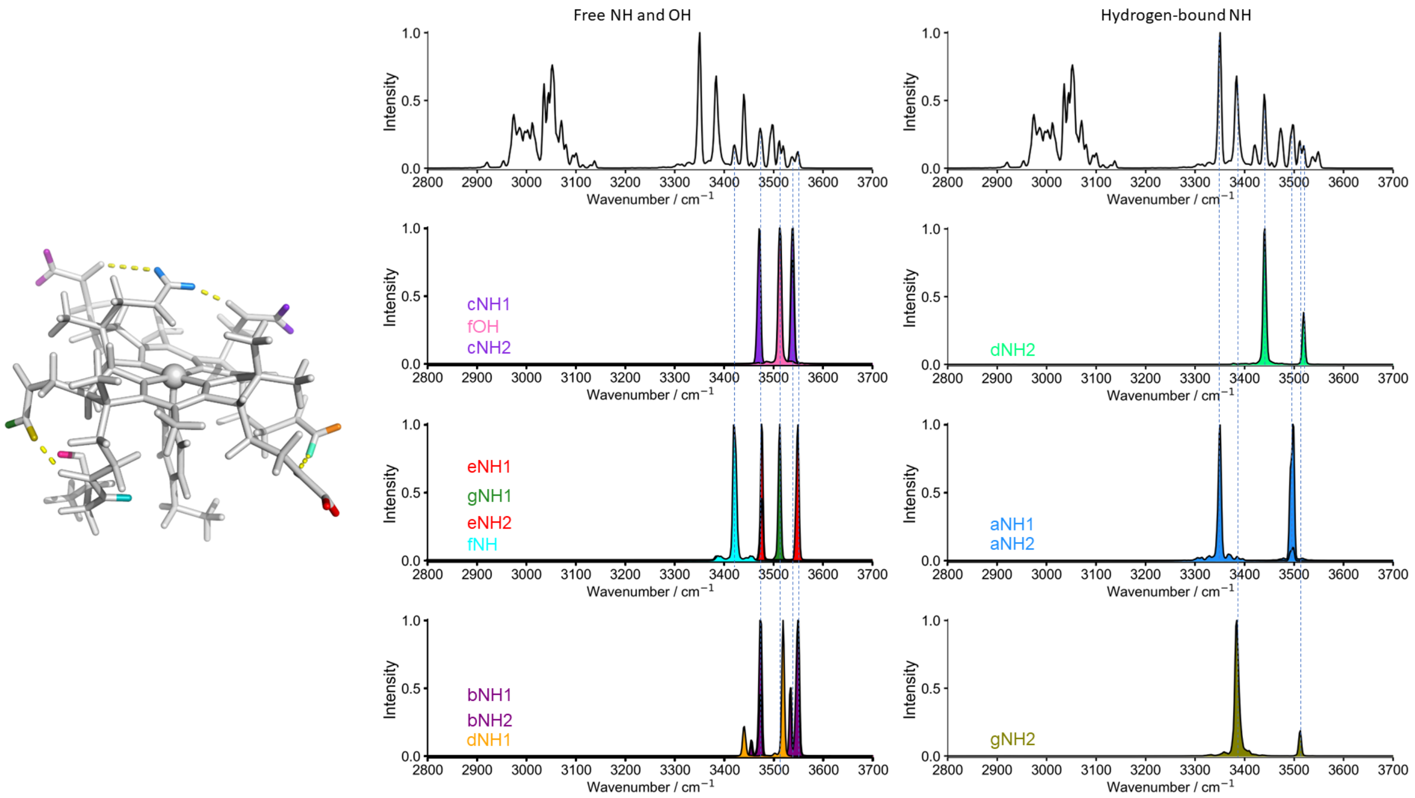

Figure S5: Spectra calculated via FT-DAC averaged over fifty MD trajectories for α-4-EtPhCbi at 50 K, together with spectra extracted for individual free N-H and O-H bonds (left) as well as hydrogen-bound N-H bonds (right).

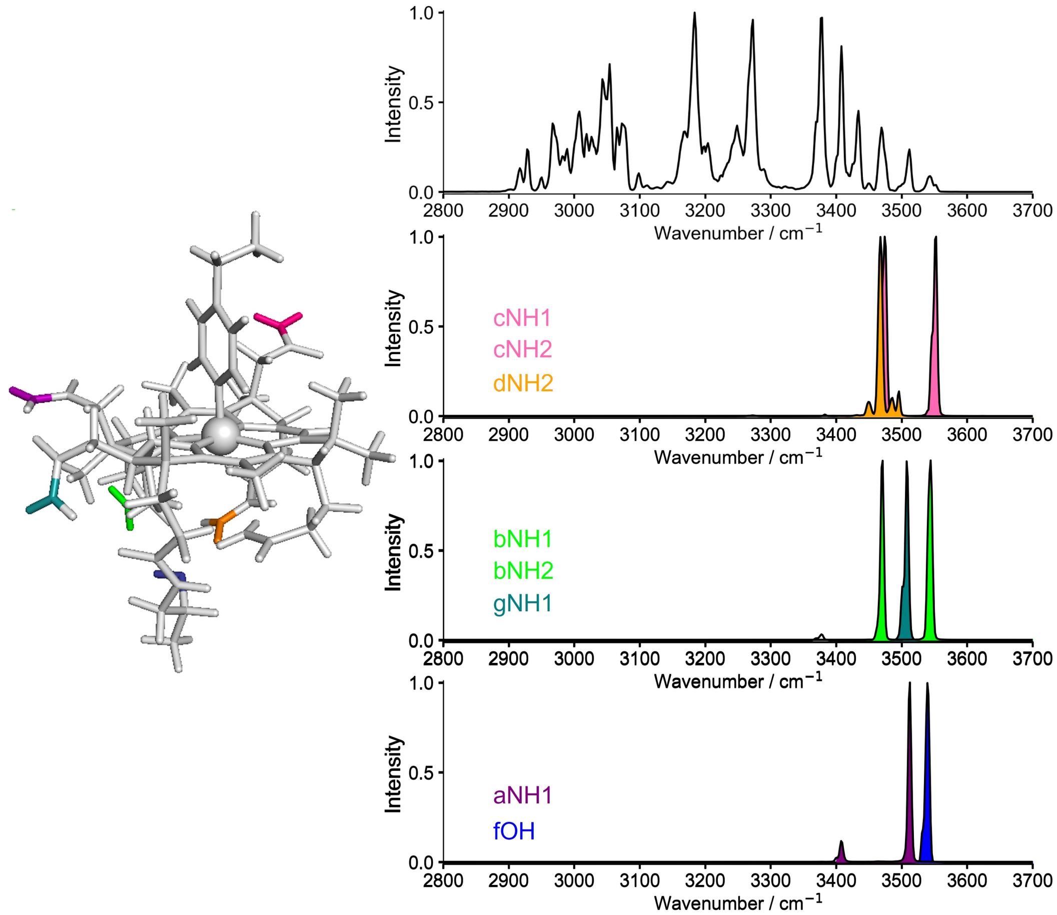

Figure S6: Spectra calculated via FT-DAC averaged over fifty MD trajectories for βEtPhCbi at 50 K, together with spectra extracted for individual free N-H and O-H bonds.

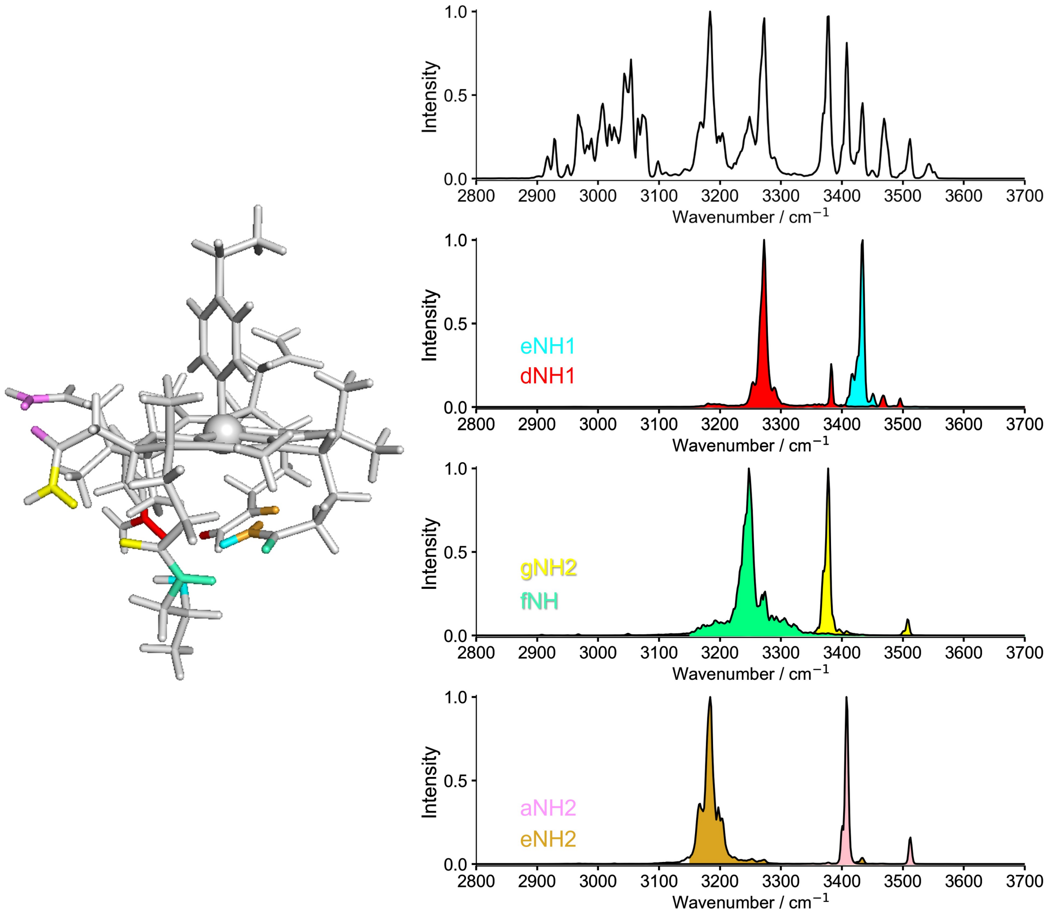

Figure S7: Spectra calculated via FT-DAC averaged over fifty MD trajectories for βEtPhCbi at 50 K, together with spectra extracted for individual hydrogen-bound N-H bonds.

S2.4 CP2K calculations

BOMD simulations with DFT as an electronic structure method were performed using CP2K program package, version 8.2.[9]

In this section, we provide an input file template for the MD calculations performed with CP2K, where <INLINE XYZ> is the placeholder for the Cartesian coordinates of the calculated molecular ion. <CH> is the placeholder for the ion's charge.The keywords related to resource management are omitted.

```
&GLOBAL
 PROJECT <NAME>
 RUN_TYPE MD
 PRINT_LEVEL LOW
&END GLOBAL

```

```
&FORCE_EVAL
 &DFT
   CHARGE <CH>
   BASIS_SET_FILE_NAME  BASIS_MOLOPT
   POTENTIAL_FILE_NAME  GTH_POTENTIALS
  &MGRID
   CUTOFF 280
   REL_CUTOFF 40
   NGRIDS 5
  &END
  &SCF
    SCF_GUESS ATOMIC
    MAX_SCF 200
    &OT
     MINIMIZER DIIS
     PRECONDITIONER FULL_SINGLE_INVERSE
    &END
    &PRINT
      &RESTART
        &EACH
          MD 0
        &END
      &END
     &END
    &END
   &LOCALIZE
     METHOD CRAZY
     MAX_ITER 2000
     &PRINT
       &WANNIER_CENTERS
         IONS+CENTERS
         FILENAME =<NAME.xyz>
         &EACH
           MD 5
         &END
       &END
      &END
    &END
    &XC
      &XC_FUNCTIONAL BP
      &END
      &XC_GRID
```

```
        XC_DERIV NN10_SMOOTH
        XC_SMOOTH_RHO NN10
      &END
      &VDW_POTENTIAL
         DISPERSION_FUNCTIONAL PAIR_POTENTIAL
        &PAIR_POTENTIAL
          TYPE DFTD3
          PARAMETER_FILE_NAME dftd3.dat
          REFERENCE_FUNCTIONAL BP86
        &END
      &END
     &END
   &END
   &SUBSYS
   &CELL
     ABC 20.0 20.0 20.0
   &END
   &COORD
   <INLINE XYZ>
   &END
   &KIND H
     BASIS_SET DZVP-MOLOPT-GTH
     POTENTIAL GTH-BP-q1
   &END
   &KIND O
     BASIS_SET DZVP-MOLOPT-GTH
     POTENTIAL GTH-BP-q6
   &END
   &KIND C
     BASIS_SET DZVP-MOLOPT-GTH
     POTENTIAL GTH-BP-q4
   &END
   &KIND N
     BASIS_SET DZVP-MOLOPT-GTH
     POTENTIAL GTH-BP-q5
   &END
   &KIND Co
     BASIS_SET DZVP-MOLOPT-SR-GTH
     POTENTIAL GTH-BP-q17
   &END
 &END
&END
```

```
&MOTION
 &MD
   ENSEMBLE NVT
   STEPS 40000
   TIMESTEP 0.5
   &THERMOSTAT
     TYPE NOSE
     &NOSE
       TIMECON 100
     &END
    &END
    TEMPERATURE 50
 &END
 &PRINT
   &RESTART
     &EACH
       MD 1
     &END
    &END
 &END
&END
```

The BOMD spectra calculated without the D3 correction are shown in Figure S8.

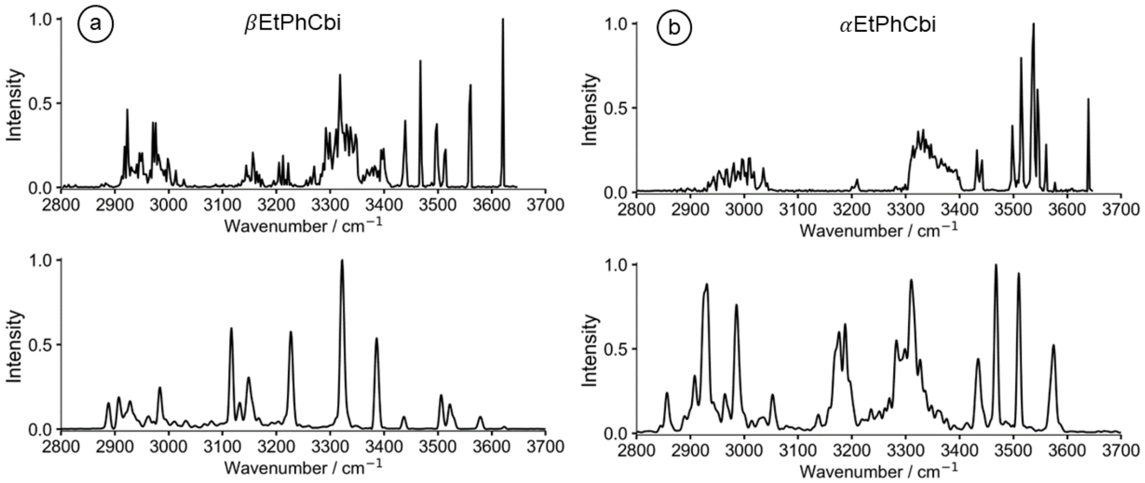

Figure S8: Comparison of the experimental CIVP spectra of a) β- and b) α-4-EtPhCbi with the calculated BOMD IR spectra using BP86/MOLOPT-DZVP-SR-GTH method.

To allow better comparison between the harmonic and MD simulations of IR spectra, harmonic spectra were calculated using CP2K package as well, with the same method/basis set combination used for the MD simulations. The resulting comparison is shown in Figure S9.

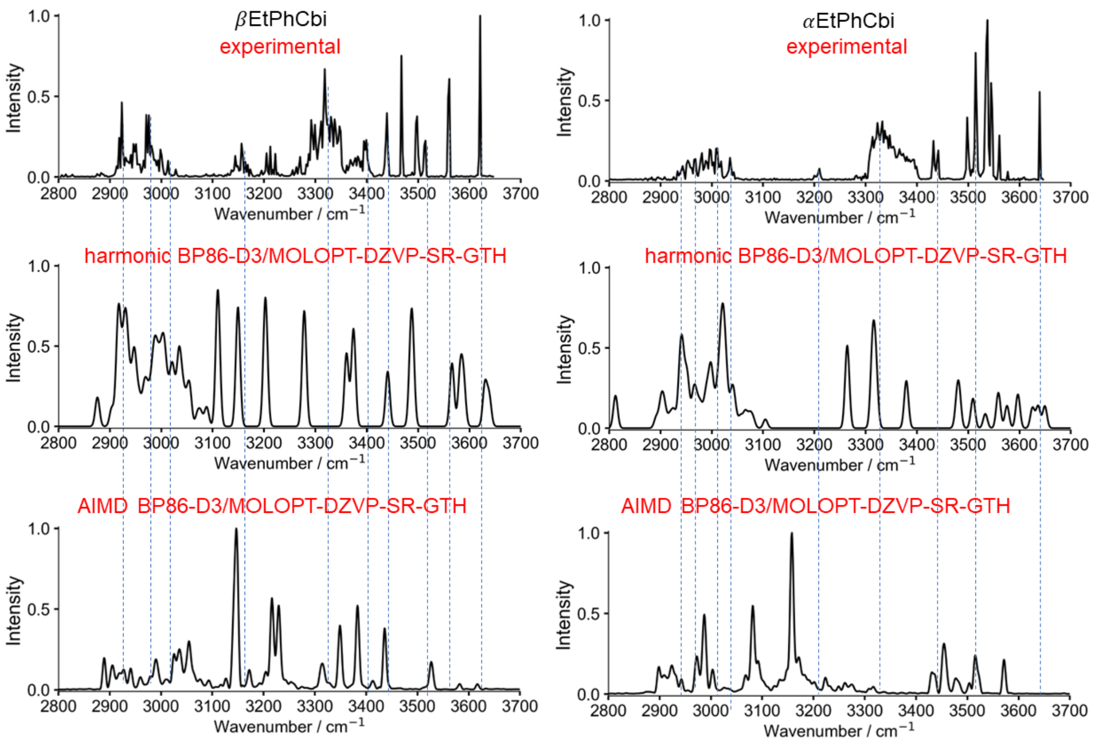

Figure S9: Comparison of the experimental CIVP spectra of a) β- and b) α-4-EtPhCbi with the calculated harmonic IR spectra using BP86-D3/MOLOPT-DZVP-SR-GTH method, as well as the BOMD spectra produced with the same method.

S3 Experimental evaluation of tag effect

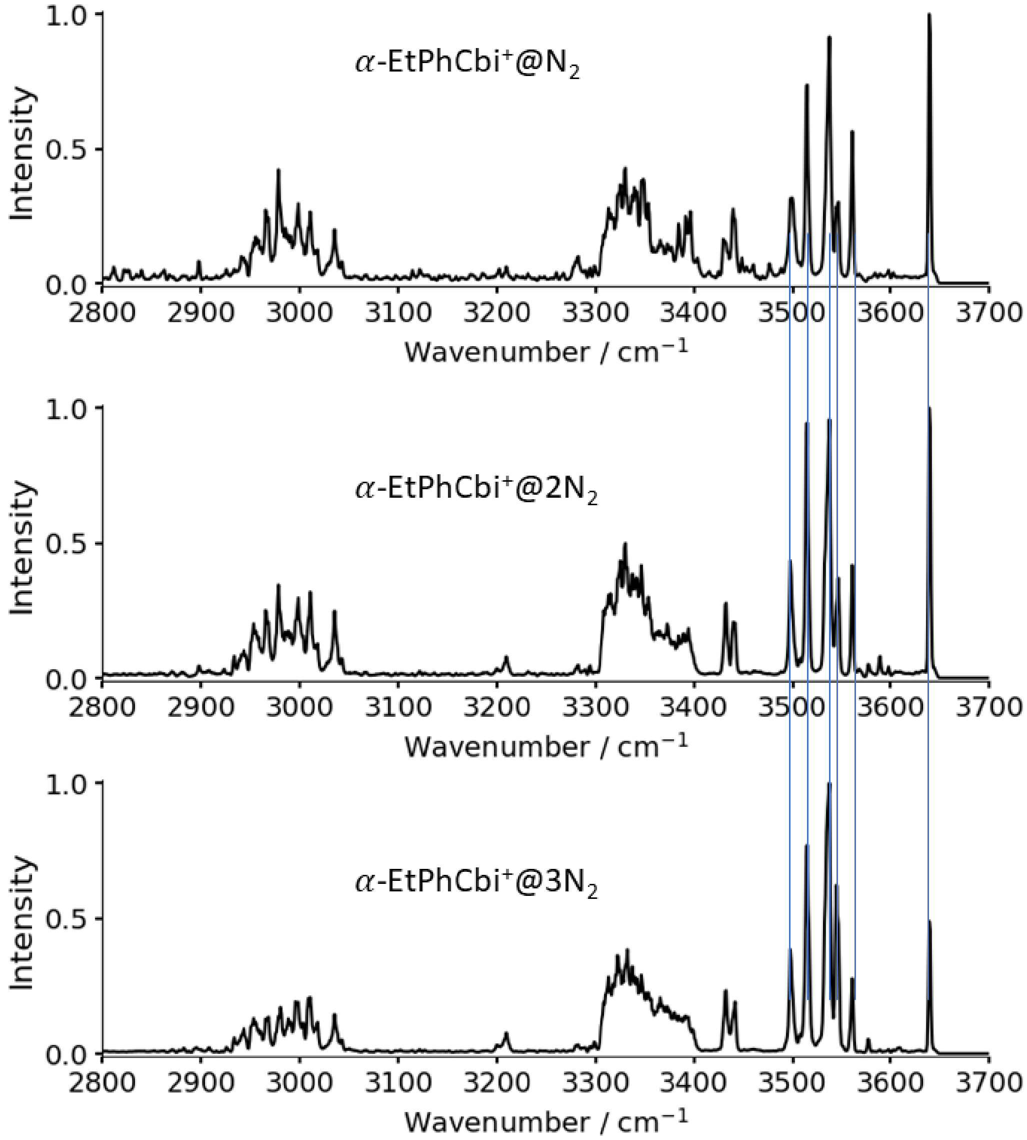

Figure S10: Comparison of the experimental CIVP spectra α-4-EtPhCbi recorded with different number of N_2 tags.

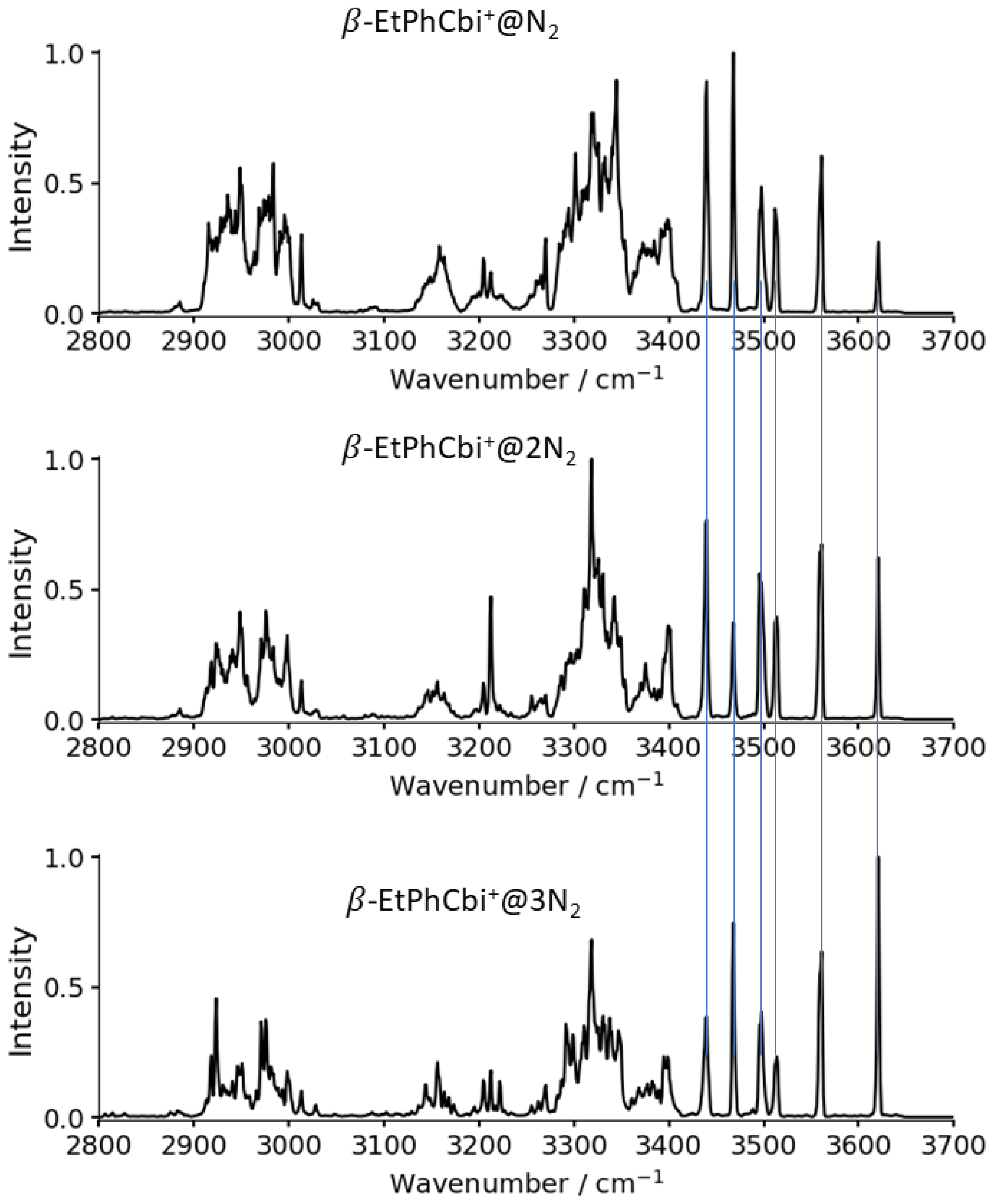

Figure S11: Comparison of the experimental CIVP spectra β-4-EtPhCbi recorded with different number of N_2 tags.

References

(1) Zheng, G.; Sonnenberg, L.; Hada, M.; Ehara, M.; Toyota, K.; Fukuda, R.; Hasegawa, J.; Ishida, M.; Nakajima, T.; Honda, Y., et al. *Gaussian Inc., Wallingford CT* **2009**.

(2) Larsen, A. H.; Mortensen, J. J.; Blomqvist, J.; Castelli, I. E.; Christensen, R.; Dułak, M.; Friis, J.; Groves, M. N.; Hammer, B.; Hargus, C., et al. *Journal of Physics: Condensed Matter* **2017**, *29*, 273002.

(3) Bannwarth, C.; Ehlert, S.; Grimme, S. *Journal of chemical theory and computation* **2019**, *15*, 1652–1671.

(4) Li, Z.; Zadoyan, R.; Apkarian, V.; Martens, C. *The Journal of Physical Chemistry* **1995**, *99*, 7453–7465.

(5) Wang, H.; Agmon, N. *The Journal of Physical Chemistry A* **2016**, *120*, 3117–3135.

(6) Tsybizova, A.; Paenurk, E.; Gorbachev, V.; Chen, P. *The Journal of Physical Chemistry A* **2020**, *124*, 8519–8528.

(7) Thomas, M.; Brehm, M.; Fligg, R.; Vöhringer, P.; Kirchner, B. *Physical Chemistry Chemical Physics* **2013**, *15*, 6608–6622.

(8) MD4IR. https://gitlab.ethz.ch/paenurke/md4ir (Accessed 2023-02-16).

(9) Kühne, T. D.; Iannuzzi, M.; Del Ben, M.; Rybkin, V. V.; Seewald, P.; Stein, F.; Laino, T.; Khaliullin, R. Z.; Schütt, O.; Schiffmann, F., et al. *The Journal of Chemical Physics* **2020**, *152*, 194103.
